# Does clavicular shortening after nonoperative treatment of midshaft fractures affect shoulder function? A systematic review

**DOI:** 10.1007/s00402-017-2734-7

**Published:** 2017-06-21

**Authors:** Sarah Woltz, Alysia Sengab, Pieta Krijnen, Inger B. Schipper

**Affiliations:** Department of Trauma Surgery, Leiden University Medical Center, P.O. Box 9600, 2300 RC Leiden, The Netherlands

**Keywords:** Clavicular shortening, Shoulder function, Nonoperative treatment, Malunion

## Abstract

**Introduction:**

Clavicular shortening due to non-anatomical healing of displaced clavicular fractures is believed to have a negative effect on shoulder function after recovery. The evidence for this, however, is equivocal. This review aimed to systematically evaluate the available literature to determine whether the current beliefs about clavicular shortening can be substantiated.

**Materials and methods:**

This systematic review was performed following the Preferred Reporting Items for Systematic reviews and Meta-Analyses (PRISMA) statement. PubMed, EMBASE, Web of Science and the Clinical Trial Registry were searched to identify all studies published in English that evaluated the association between clavicular shortening and shoulder function in patients aged ≥16 years with a nonoperatively treated, displaced midshaft clavicular fracture. Relevant data from the selected studies was extracted and summarized. Risk of bias of the included studies was assessed using the MINORS instrument.

**Results:**

Six studies, of which five were retrospective, were included in this review analyzing a total of 379 patients. Due to heterogeneity in methods and reporting across studies, a pooled analysis of the results was not feasible. No clear associations were found between shortening and shoulder function scores (DASH and Constant score) or arm strength in each of the included studies.

**Conclusion:**

The existing evidence to date does not allow for a valid conclusion regarding the influence of shortening on shoulder function after union of nonoperatively treated midshaft clavicular fractures. Shortening alone is currently not an evidence-based indication to operate for the goal of functional improvement. Well-powered prospective comparative studies are needed to draw firm conclusions.

**Electronic supplementary material:**

The online version of this article (doi:10.1007/s00402-017-2734-7) contains supplementary material, which is available to authorized users.

## Introduction

Midshaft fractures of the clavicle are common and often displaced [1, 2]. Treatment of these fractures is aimed at a complete recovery of the shoulder function, especially in younger patients. In nonoperatively treated patients, closed reduction of the fracture is difficult to achieve and to maintain, and is therefore no longer attempted [3, 4]. A certain degree of clavicular shortening often remains after union due to overlap of the fracture fragments, caused by traction of the pectoral and deltoid muscles and the weight of the arm that pull the lateral fragment ventro-caudally and medially, while the sternocleidomastoid muscle pulls the medial fragment upwards and dorsally [5].

In addition to the historic indications for operative fixation of displaced clavicular fractures (i.e., open fracture, neurovascular compromise and compromised skin), evidence-based reasons for operative fixation include reduction of the risk of nonunion and a quicker recovery [2, 6–8]. Substantial shortening of the clavicle is also considered to be an indication for operative treatment, partly because it may increase the risk of nonunion [9, 10], but also because shortening is thought to lead to a poorer functional outcome after fracture union. It is believed that the significant changes in the position of the glenoid fossa and shoulder girdle, and winging of the scapula after shortening of the clavicle are responsible [4, 11–13]. Also, muscle balance and tension can be reduced if the clavicle is shortened [12]. This altered anatomy may result in the sequelae that have been reported after nonoperative treatment [4, 9]. Recent comparative studies, however, have not demonstrated a functional benefit for healed fractures after restoration of the anatomy with operative fixation compared with nonoperative treatment [7, 8].

It is important to clarify whether there is sufficient evidence to support the assumption that shortening is an indication for surgery to improve the functional outcome. Studies that have evaluated this relationship, however, show inconsistent results. While some reported that a larger shortening causes more complaints, pain and dissatisfaction [9, 14, 15], others found no association between shortening and sequelae [16–18]. These studies, however, did not clearly evaluate an association with the function of the shoulder.

The aim of this review, therefore, was to summarize the available literature to evaluate whether clavicular shortening is negatively associated with shoulder function (i.e., patient-reported function, range of motion or arm strength) at latest follow-up after nonoperative treatment.

## Materials and methods

This systematic review was performed according to the ‘Preferred Reporting Items for Systematic reviews and Meta-Analyses: the PRISMA statement’ [19].

### Search strategy and eligibility criteria

The literature search was performed in Pubmed, Embase, Web of Science and the Clinical Trial Registry in December 2016. The search strategy was composed by an experienced medical librarian and combined various synonyms of the keywords ‘clavicle’, ‘fracture’, ‘midshaft’, ‘nonoperative’ and ‘shortening’ (see Supplementary Appendix 1 for the full search strategy).

Studies were eligible if they (1) included patients older than 15 years of age with a nonoperatively treated, displaced midshaft clavicular fracture, (2) evaluated the association between the extent of clavicular shortening and function of the shoulder (i.e., patient-reported functional outcome, range of motion and/or arm strength), and (3) were written in English.

Articles were excluded if they (1) included less than 20 patients, or (2) also analyzed medial and/or lateral clavicular fractures and the results for midshaft fractures were not reported separately. No date range was specified.

After removal of duplicates, the title and abstract of the identified articles were independently screened for eligibility by the first two authors. The full-text articles of the potentially relevant studies were read and judged for eligibility. The reference lists of these articles were searched for additional relevant studies, which were included if the above mentioned inclusion criteria applied. Disagreements were resolved by discussion.

### Data extraction

From each included article, data were extracted by the first two authors, including study characteristics (study design, number of included patients and duration of follow-up) and patient characteristics (age, gender and type of nonoperative treatment). Outcomes of interest were clavicular shortening and shoulder function (measured by means of the DASH-score [20], Constant score [21], arm strength and/or range of motion), and the reported association between shortening and function. A meta-analysis could not be performed because there was considerable variation in the definitions of shortening and the statistical methods across studies.

### Quality assessment

Methodological quality of the included studies was independently assessed by the first two authors using the “Methodological Index for Non-Randomized Studies” (MINORS) instrument, which consists of eight items regarding the design of non-comparative studies [22]. Each item is appointed a score (“0” = not reported; “1” = reported but inadequate; “2” = reported and adequate) with an optimal total score of 16.

## Results

### Literature search

**Fig. 1:**
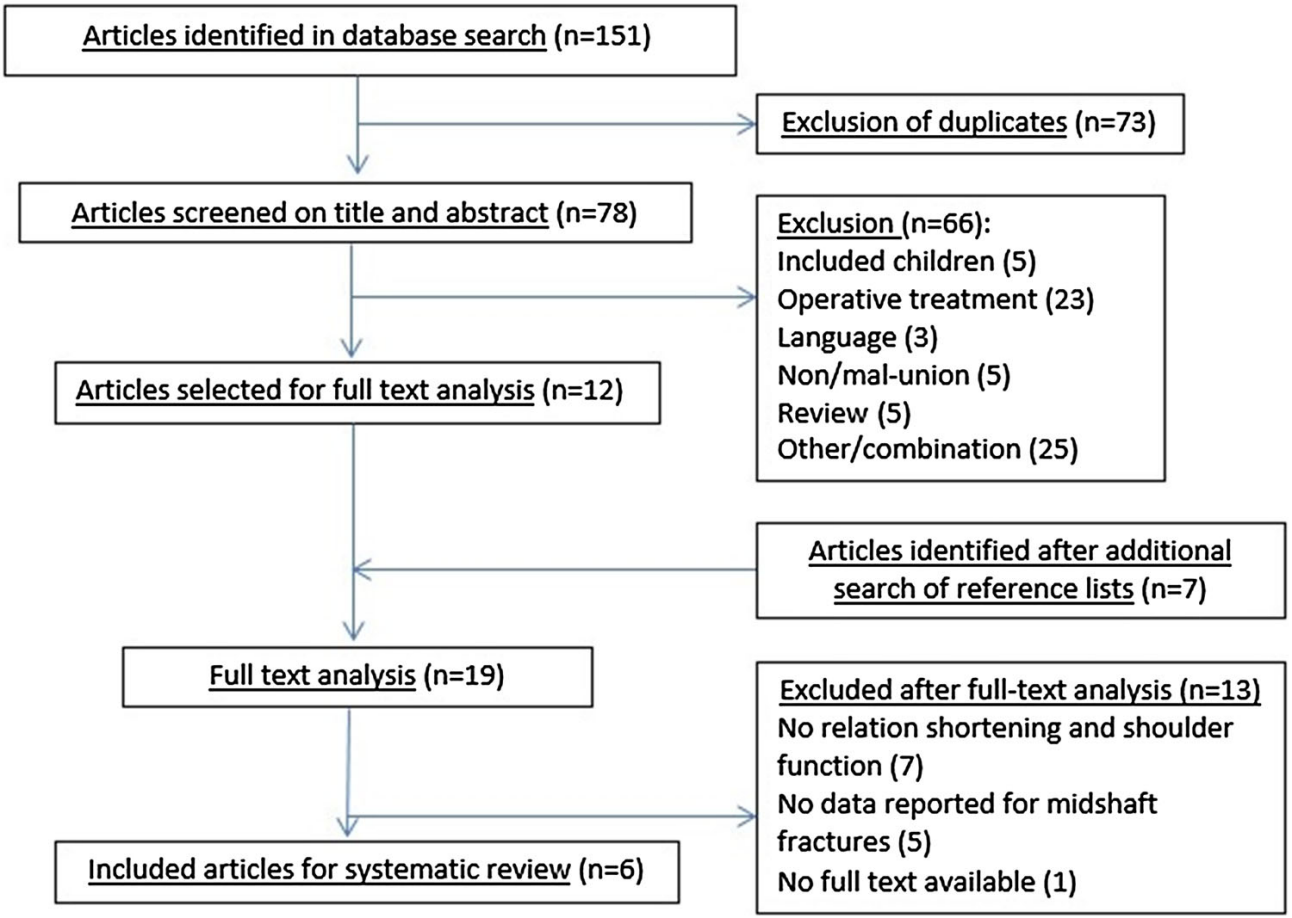
Flowchart of the included articles

The search in Pubmed, Web of Science, Embase and the Clinical Trial Registry identified 151 potentially eligible articles. After removal of duplicates, 78 articles were screened based on title and abstract, of which 12 were selected. Screening the reference lists yielded another 7 potentially relevant articles. After reading the full text of these 19 articles, 6 articles were included in this systematic review based on the selection criteria (Fig. 1).

### Study characteristics

The included studies were published between 2006 and 2015, and evaluated a total of 379 patients (Table 1). Five studies were retrospective [5, 23–26] and one was prospective [27]. In four studies, determining the relationship between shortening and shoulder function was the primary study aim [23, 24, 26, 27]. Follow-up was at least 12 months in all studies, with a frequency-weighed mean of 4.5 years. Most patients were immobilized with a sling or figure-of-eight bandage for various time periods. The prospective study reported a loss to follow-up of 8.5% [27].

**Table 1:** Characteristics of the included studies and patients analyzed

References	Study design	No. of evaluated patients^a^	Mean time since trauma, months	Mean age, years (SD)	Male (%)	Type of nonoperative treatment
Fuglesang et al. [26]	Retrospective	59/92	32 (12–59)	39.1 (12.3)	83	Sling
Figueiredo et al. [27]	Prospective	54/59	12	34 (13)	81	FEB + PT
Stegeman et al. [23]	Retrospective	32/74	12–72	Median 31 (range 21–62)	84	Not reported
Rasmussen et al. [24]	Retrospective	136/237	55 (24–83)	35 (15)	79	FEB (*n* = 50), simple sling (*n* = 70), C&C (*n* = 13), no support (*n* = 3)
Postacchini et al. [25]	Retrospective	68/119^b^	104	36.9	65	Sling or FEB
McKee et al. [5]	Retrospective	30/63	55 (12–72)	37	73	Sling

*SD* standard deviation, *FEB* figure of eight bandage, *C&C* collar and cuff, *PT* physiotherapy

^a^No of evaluated patients/no of eligible patients (or included patients for prospective study)

^b^119 patients were eligible for inclusion in total. Number of eligible patients with Allman type 1b/c fracture not stated

### Clavicular shortening

The studies expressed clavicular shortening in different ways; either by measuring the difference in length between the injured and the contralateral clavicle [5, 24, 27], or by measuring the overlap of fracture fragments [26]. Stegeman and Postacchini additionally calculated the proportional shortening by dividing the overlap of fracture fragments by the sum of the length of the injured clavicle and the measured overlap [23, 25]. Shortening was also measured at different time points: on the index trauma radiographs [24–27], or on radiographs taken after the fracture had united [5, 23].

The reported mean shortening (Table 2) ranged from 9.2 mm (SD 6.4) to 25 mm (SD 16). Three studies compared patients with a shortening less than 20 mm with those having 20 mm or more shortening. In these studies, 15, 37 and 19% of the study population had a shortening of ≥20 mm [5, 24, 27]. Fuglesang used the median shortening of 15 mm as cut-off value to determine small or large shortening, thus creating two equally sized groups [26].

**Table 2:** Relation between clavicular shortening and Constant score and/or DASH score

References	Mean shortening in mm (SD)	Mean Constant score (SD)	Mean DASH score (SD)	Correlation (*r*) or *p* value
Fuglesang et al. [26]	17.1 (7.1)	81 (69–90) (median)	6.7 (0.8–19) (median)	
	<15 mm: *n* ≈ 30	80 (64–88)	7 (3–27)	*p* = 0.5 (constant)
	>15 mm: *n* ≈ 30	84 (74–90)	7 (0–11)	*p* = 0.1 (DASH)
Figueiredo et al. [27]	9.2 (6.4)	N/A	3.38 (9.21)	*r* = −0.017; *p* = 0.90
	<20 mm: *n* = 47 (81%)		3.38 (CI 9.56)	*p* = 0.53
	>20 mm: *n* = 11 (19%)		3.33 (CI 7.02)	
Rasmussen et al. [24]	11.6 (8.2)	86.3 (29–100)	N/A	*r* = 0.14; *p* > 0.05
	<20: *n* = 116 (85%)	7.2 (10.3)^a^		*p* = 0.79
	>20: *n* = 20 (15%)	7.9 (10.3)		
Postacchini et al. [25]	Males: 14.1 (8.9); 8.9% (5.6%)^b^	Allman 1B^c^: 87.1 Allman 1C: 85.6	N/A	
	Females: 10.9 (7.8); 8.3% (6.0%)^1^	CS ≥ 90 (*n* = 55): 7.7% CS ≤ 80 (*n* = 9): 13.2%		*p* < 0.05
McKee et al. [5]	14.5 (8.6)	71 (SD not given)	24.6 (SD not given)	*r* = −0.20; *p* = 0.44 *r* = 0.32; *p* = 0.11
	<20 mm: *n* = 19 (63%)			*p* = 0.06
	≥20 mm: *n* = 11 (37%)		DASH > 30 points: 3/19 (16%) 7/11 (64%)	

^a^Mean difference in Constant score between injured and uninjured shoulder

^b^Proportional shortening: overlap of fracture fragments divided by sum of overlap and length of injured clavicle

^c^Allman type 1B: displaced fractures, Allman type 1C: displaced with third bone fragment

### Shoulder function

Various outcome measures were used to evaluate shoulder function at final follow-up. Mean DASH scores ranged from 3.38 to 24.6 in four studies (Table 2) [5, 23, 26, 27]. The mean Constant score (Table 2) was reported in five studies (range 71–96) [5, 23–26]. McKee found much poorer mean Constant and DASH scores than the other studies, and both functional scores were significantly worse than the normative value for the general population (71 vs 92 and 24.6 vs 10.1, respectively) [5, 28, 29]. One study that compared the injured with the healthy shoulder, reported a significant difference in Constant score (86.3 vs 93.7, *p* < 0.001) [24], whereas Constant and DASH scores of the patients in another study were similar to those of matched controls [23].

Strength was measured by Stegeman and McKee with a hand-held dynamometer and with the Baltimore Therapeutic Equipment (BTE) Work Simulator, respectively [5, 23]. Whereas Stegeman found no significant mean differences in strength compared with the contralateral shoulder for six different motions, McKee reported that the injured shoulder had 81–85% of the strength and 67–82% of the endurance of the patients’ uninjured shoulder (*p* < 0.05 for all motions) (Table 3).

**Table 3:** Relation between clavicular shortening and shoulder strength

References	Mean shortening in mm (SD)	Mean strength in Newton (95% CI)	Correlation or *p* value
Stegeman et al. [23]	25 (16)	Adduction: 7.2 (−3.5 to 18)^b^	*β* = − 1.29 (*p* = 0.07)
	13% (8%)^a^	Abduction: −0.1 (−8.8 to 8.6)	*β* = − 0.47 (*p* = 0.4)
		Anteflexion: 9.6 (−3.1 to 22)	*β* = 0.59 (*p* = 0.5)
		Retroflexion: 14.6 (−6.7 to 9.8)	*β* = − 0.08 (*p* = 0.9)
		Exorotation: 2.0 (−3.2 to 7.3)	*β* = 0.08 (*p* = 0.8)
		Endorotation: 5.1 (−0.8 to 11.1)	*β* = 0.37 (*p* = 0.3)
McKee et al. [5]	14.5 (8.6	Flexion: 81%, 75%^c^	ns
		Abduction: 82%, 67%	*r* = −0.32 (*p* = 0.06)
	<20: *n* = 19 (63%)	Exorotation: 81%, 82%	ns
	≥20: *n* = 11 (37%)	Endorotation: 85%, 78%	ns

^a^Proportional shortening: overlap of fracture fragments divided by sum of overlap and length of injured clavicle

^b^Difference in strength between uninjured and injured shoulder. *p* > 0.05 for all comparisons

^c^Strength and endurance of injured shoulder as a percentage of the uninjured shoulder

Three studies reported the range of motion of the injured and contralateral shoulders but did not analyze its association with clavicular shortening [5, 23, 25]. For this reason, results on range of motion are not included in this review.

### Association between shortening and shoulder function

The association between clavicular shortening and the DASH score was analyzed in three studies. Results are presented in Table 2. No statistically significant linear correlations were found [5, 27]. Also, no difference in DASH scores existed between patient groups when shortening was dichotomized using cut-off values of 15 mm [26] or 20 mm [27].

McKee reported that among patients with ≥20 mm shortening, a poor DASH score of >30 seemed more prevalent than among patients with <20 mm shortening (64 vs 16%, *p* = 0.06) [5].

Four articles reported on the association between shortening and Constant score (Table 2) [5, 24–26]. No linear relationship was found [5, 24]. Also, a larger shortening (more than 20 or 15 mm) did not result in a significantly lower Constant score [24, 26]. Only Postacchini found that shortening was significantly larger in patients with a Constant score below 80, than in patients with a Constant score of 90 or higher [25]. Stegeman reported that all DASH and Constant scores were in the normal range of values, and therefore, did not analyze a relation with shortening [23].

Two studies evaluated arm strength (Table 3) [5, 23]. Only the association between shortening and abduction endurance approached statistical significance in one study [5]. There was no relation between shortening and endurance for all other motions, nor with strength [5, 23].

### Risk of bias and quality assessment

Table 4 shows the results for the assessment of the methodological quality for each study. All studies had a clear aim and collected appropriate data according to a beforehand established protocol. In most studies, however, there was risk of observer bias because function scores and shortening were measured by the same researcher, and of selection bias because only a portion of the eligible patients participated. Only one study mentioned the intended sample size, but no calculation or rationale was stated [23].

**Table 4:** Methodological quality of included studies assessed according to the Methodological Index for Non-Randomized Studies (MINORS) instrument [20]

	Fugle-sang et al. [26]	Figueiredo et al. [27]	Stege-man et al. [23]	Rasmussen et al. [24]	Postac-chini et al. [25]	McKee et al. [5]
1. A clearly stated aim	2	2	2	2	2	2
2. Inclusion of consecutive patients	1	2	1	1	1	1
3. Prospective collection of data^a^	2	2	2	2	2	2
4. Endpoints appropriate to the aim of the study	2	2	2	2	2	2
5. Unbiased assessment of the study endpoint	0	0	0	0	1	1
6. Follow-up period appropriate to the aim of the study	2	2	2	2	2	2
7. Loss to follow-up less than 5%	2	1	2	2	2	2
8. Prospective calculation of the study size	0	0	1	0	0	0
Total	11	11	12	11	12	12

The items are scored 0 (not reported), 1 (reported but inadequate) or 2 (reported and adequate). Maximum score is 16

^a^Data were collected according to a protocol established before the beginning of the study

## Discussion

In daily practice, shortening of a midshaft clavicular fracture is often regarded as a risk factor for functional impairment after fracture union. This review of the available literature included six studies and showed that there is not enough evidence to substantiate this assumption. Therefore, shortening of a fractured clavicle should currently not be regarded as an evidence-based indication to operate for the goal of functional improvement. In a clear evidence-supported approach, other indications should be considered such as the reduced risk of nonunion and earlier functional recovery. Also, following the principles of shared decision making, patients’ preferences could be reason to opt for surgical treatment.

A difficulty in studying possible influences on shoulder function is that Constant and DASH scores are generally in the upper range of the scale after clavicular fractures. Due to this ceiling-effect subtle differences in scores remain undetected, although such small differences in scores are unlikely to be clinically relevant for most patients. Also, the number of patients with a large amount of shortening in the included studies was low. For instance, the association that was found between a larger shortening and a Constant score below 80 in one study, was based on only nine patients [25].

The most important limitation of this review is the heterogeneity in methods and definitions across studies. The research groups obviously differed in their ideas about the best way to measure clavicular shortening. Most conspicuous are the different time points at which shortening was measured; either directly after the injury, or after fracture union. Fuglesang reported that the median difference in clavicular length between initial and final radiographs was 7.5 mm (25th–75th percentiles 4–10), and that there were large individual adjustments suggesting that the final amount of shortening cannot be reliably predicted on initial radiographs [26]. Two previous studies by Smekal et al., however, showed no significant difference between initial and final proportional shortening [5.4 (SD 4.0) vs 4.7 (SD 3.9), *p* = 0.16; and 5.0 (SD 3.3) vs 5.1 (SD 3.5), *p* = 0.86] [30, 31].

Also, different techniques were applied to measure shortening. Three studies used the length of the contralateral clavicle, assuming that the clavicles had been equally long before fracture [5, 24, 27]. It is, however, well known that a considerable asymmetry of both clavicles may exist within individuals: a mean difference in clavicular length of 4.25 mm (SD 3.8) and an asymmetry of ≥5 mm in 28.5% of uninjured, skeletally mature adults has been reported [32].

In addition, four of the studies expressed shortening as the absolute difference in clavicular length [5, 24, 26, 27]. A large absolute shortening, however, potentially has more influence on shoulder kinematics in a patient with a short clavicle than in a tall patient with a long clavicle [33]. Stegeman and Postacchini accounted for these issues by expressing shortening as a proportion of the clavicular length, and using the estimated length of the original bone instead of the contralateral clavicle for comparison [23, 25].

In summary, the existing evidence to date does not allow for a valid conclusion regarding the influence of shortening on shoulder function after union of nonoperatively treated midshaft clavicular fractures. Shortening alone is currently not an evidence-based indication to operate for the goal of functional improvement. Well-designed prospective studies including sufficient numbers of patients with a substantial amount of shortening are needed to formulate a conclusion.

## Electronic supplementary material

Below is the link to the electronic supplementary material.

Supplementary material 1 (DOCX 12 kb)
