# Supplementary material for: Does clavicular shortening after nonoperative treatment of midshaft fractures affect shoulder function? A systematic review
Source: Arch Orthop Trauma Surg. 2017 Jun 21;137(8):1047–53. doi: 10.1007/s00402-017-2734-7 (PMC5511301; doi:10.1007/s00402-017-2734-7)
Supplement: Supplementary file 1 — Supplementary material 1 (DOCX 12 kb) [file 402_2017_2734_MOESM1_ESM.docx]

**Appendix 1 Search strategies**

Pubmed: ("Clavicle"[Mesh] OR "Clavicle"[tw] OR "clavicular"[tw] OR "clavicula"[tw]) AND ("fractures, bone"[MeSH] OR "fractures"[tw] OR "fracture"[tw]) AND ("midshaft"[tw] OR "mid-shaft"[tw] OR "mid shaft"[tw] OR "middle third"[tw] OR "middle-third"[tw]) AND ("Shortening"[tw] OR "Shortenings"[tw] OR "shortened"[tw])  AND ("conservative"[tw] OR "conservatively"[tw] OR "nonoperative"[tw] OR "nonoperatively"[tw] OR "non-operative"[tw] OR "non-operatively"[tw] OR "nonsurgical"[tw] OR "nonsurgically"[tw] OR "non-surgical"[tw] OR "non-surgically"[tw] OR "sling"[tw] OR "immobilisation"[tw] OR "immobilization"[MeSH Terms] OR "immobilization"[tw] OR "bandages"[MeSH] OR "bandages"[tw] OR "bandage"[tw])

Embase: (((exp "Clavicle"/ OR "Clavicle".mp. OR "clavicular".mp. OR "clavicula".mp.) AND (exp "fracture"/ OR "fractures".mp. OR "fracture".mp.)) OR exp clavicle fracture/) AND ("midshaft".mp. OR "mid-shaft".mp. OR "mid shaft".mp. OR "middle third".mp. OR "middle-third".mp.) AND ("Shortening".mp. OR "Shortenings".mp. OR "shortened".mp.)  AND (exp conservative treatment/ OR "conservative".mp. OR "conservatively".mp. OR "nonoperative".mp. OR "nonoperatively".mp. OR "non-operative".mp. OR "non-operatively".mp. OR "nonsurgical".mp. OR "nonsurgically".mp. OR "non-surgical".mp. OR "non-surgically".mp. OR "sling".mp. OR "immobilisation".mp. OR exp fracture immobilization/ OR "immobilization".mp. OR exp bandage/ OR "bandages".mp. OR "bandage".mp.)

Web of Science: TS=(("Clavicle" OR "clavicular" OR "clavicula") AND ("fractures" OR "fracture") AND ("midshaft" OR "mid-shaft" OR "mid shaft" OR "middle third" OR "middle-third")) AND TS=("Shortening" OR "Shortenings" OR "shortened")  AND TS=("conservative" OR "conservatively" OR "nonoperative" OR "nonoperatively" OR "non-operative" OR "non-operatively" OR "nonsurgical" OR "nonsurgically" OR "non-surgical" OR "non-surgically" OR "sling" OR "immobilisation" OR "immobilization" OR "bandages" OR "bandage")

Clinical Trial Register: "Clavicle" AND "fractures" AND ("mid-shaft" OR "middle third") AND "Shortening"
